# A Comprehensive Review on Innovative Food Gelling Strategies for Sustainable Production of Meat Analogs and Restructured Meat

**DOI:** 10.3390/gels12020147

**Published:** 2026-02-05

**Authors:** AMM Nurul Alam, Abdul Samad, Ayesha Muazzam, So-Hee Kim, Chan-Jin Kim, Young-Hwa Hwang, Seon-Tea Joo

**Affiliations:** 1 Division of Applied Life Science (BK21 Four), Gyeongsang National University, Jinju 52852, Republic of Korea; alam6059@yahoo.com (A.N.A.); buzdarabdulsamad@gmail.com (A.S.); ashu2nice@gmail.com (A.M.); winight123@gmail.com (S.-H.K.); ckswls090@naver.com (C.-J.K.); 2 Institute of Agriculture & Life Science, Gyeongsang National University, Jinju 52852, Republic of Korea; philoria@gnu.ac.kr

**Keywords:** meat analogs, food gels, structuring technologies, hybrid gels, 3D printing

## Abstract

The growing need for ecologically sound and ethical protein sources has contributed to the development of meat analogs (MAs) and restructured meat products (RMPs). Next generation MA and RMP production requires sustainable structuring techniques to imitate the physical, chemical, and sensory characteristics of conventional meat. Innovative gelling techniques are essential for attaining optimal texture, chewiness, and structural firmness in MAs and RMPs. Food gels can modulate water and fat retention, as well as the physical and mechanical characteristics of MA and RMP. Different gelling systems such as hydrogels, emulsion gels, oleogels, and hybrid gels contribute to texture formation, water and fat retention, juiciness, and structural integrity, which are essential for mimicking conventional meat. The role of gels as key structuring elements is integrated with advanced processing technologies such as high-moisture extrusion and 3D printing. This review discusses how protein, polysaccharide, lipid, and hybrid gelling techniques facilitate the development of MAs and RMPs with enhanced texture, sensory quality, nutritional value, and sustainability. Advanced structuring techniques, such as high-moisture extrusion, shear cell processing, and 3D printing, are explained regarding their integration of tailored gels (hydrogels, emulsion gels, oleogels, and hybrid gels) to fabricate imitated meat structures. Moreover, this article investigates the sensory and nutritional ramifications of various gelling techniques, spanning their role in juiciness and flavor composition. This review emphasizes significant research deficiencies and suggests more extensive future studies to facilitate the further development of economically viable and sustainable MAs and RMPs.

## 1. Introduction

Meat is a substantial source of high-quality protein, but its use raises concerns regarding sustainability, public health, animal welfare, economic constraints, and social and cultural taboos. The issue is exacerbated by the world’s burgeoning population, anticipated to reach an astonishing 9 billion by 2050 [1,2,3]. Recently, the scientific community has focused on converting plant proteins into fibrous products resembling meat, known as meat analogs (MAs), which are regarded as a potential and sustainable substitute for meat [4]. Another kind of product is gaining popularity, combining meat and plant ingredients into a hybrid to enhance nutritional value by retaining meat content. The processing of different ingredients (e.g., meat or vegetable protein) and their transformation into a new product with high nutritive value is known as the production of restructured meat products (RMPs) [5]. The rapid expansion of MAs [6,7,8,9] and RMPs [10,11] is reshaping the global protein market, driven by environmental concerns, public health interests, and animal welfare considerations. The global MA market has experienced exponential growth, reaching a market value of $7.9 billion by 2022 and is expected to further increase to $15.7 billion by 2027, underscoring its increasing popularity and commercial potential [12,13].

Industrial recipes use a wide range of plant-derived proteins and polysaccharides to produce MAs and RMPs that precisely imitate conventional meat in terms of texture and nutritional value. MAs can imitate the chromatic profile, structure, mechanical characteristics, taste traits, and nutritional profile of conventional meat, primarily composed of crosslinked biomaterials with additives [14]. Plant-originated proteins represent an appealing solution to these issues, as they can mimic conventional meat by creating nutritionally and structurally analogous products. These products, commonly known as MAs and RMPs, usually originate from wheat, soybeans, pea proteins, oilseed cakes, fungi, and microbial proteins. These components serve as the principal supplies for fabricating MAs and RMPs which closely mimic the nutritional composition and structural properties of conventional meat [15].

Sustainable development of the MA and RMP business requires careful consideration of standardizing fabrication techniques to utilize unconventional protein biomaterials. Moreover, sourcing inexpensive biomaterials is necessary to reduce the product cost and ensure consumer affordability [16]. A major focus should be on developing a product that very precisely imitates the texture and appearance of a real cut of meat [17]. There is an immense opportunity to incorporate bioactive compounds to enhance the health benefits of MAs and RMPs. Extensive research is required to modify extrusion and 3D printing techniques to produce products with textures similar to real meat cuts [18]. First-generation MAs and RMPs are mostly minced or processed products, such as sausages, patties, and hybrid products combined with meat recipes, which have less consumer appeal; the expectation was for a product more like a real meat [19,20]. Novel MA production techniques, such as high-moisture extruders, shear cells, and 3D printers, consume significant energy and require sophisticated instruments [21]. As a result, it is necessary to modify the techniques to ensure their sustainability and scalability. In the last few years, there has been an increasing interest in the application of food gels to conquer these challenges [19]. The principals involved in food gel preparation are easy to manage, possess minimal energy inputs, and require no complex equipment. Most often, they entail integration of plant proteins, polysaccharides, and fats to form a blend using a suitable gel-forming method [22,23].

Food gels are widely employed to simulate the semi-solid characteristics and biopolymer network structure required for MAs [12]. The manipulation of interactions among plant proteins, carbohydrates, and other food components allows the fabrication of hybrid food gels with diverse physical, chemical, and bioactive properties, thereby leading to flexibility for the application of diverse materials to fabricate and imitate the structural and taste traits of conventional meat to fulfill the consumer requirements [14]. Diversity in raw materials encompassing agricultural food waste biomaterials, plant biomaterials, mycoproteins, algae, and fungi facilitates the production of easy-to-use MAs and RMPs via gelling techniques [24]. For the sustainable incorporation of food gels in MAs and RMPs, the applied biomaterials should possess the desired characteristics for MAs and RMPs, such as moisture and fat absorption and emulsifying and foaming properties; finally, they should also be able to form gels easily [25]. These technical specifications are critical to ensure the gel-forming biomaterials notably enhance the structural, functional, and nutritional profile of MAs and RMPs [24]. Consequently, there is a booming interest in using food gels to improve the functional performance and nutritional profile of MAs. Gelling systems based on proteins, polysaccharides, lipids, or their hybrids play a central role in structuring MAs and RMPs, as they determine water-holding capacity, fat distribution, bite, and juiciness that together define meat-like quality. Innovative food gelling strategies are increasingly being used not only to mimic animal muscle structure, but also to improve sustainability by replacing animal fats, incorporating upcycled biopolymers, and enabling low-impact processing technologies [26].

Three-dimensional (3D) printing, an additive manufacturing technology, utilizes computer-aided design, software, and numerical control systems to fabricate 3D structures [27]. Presently, the 3D printing technique has found widespread applications in the processing of food gels, particularly in the manufacturing of MAs [28]. By controlling the distribution of components such as adipose tissue and the arrangement of fiber structures, 3D printing can easily produce MAs with a more refined appearance [26]. Additionally, it can meet the design requirements of various MAs by extruding raw materials from different cartridges, facilitating formulation changes, and achieving personalized customization of product texture, color, flavor, and nutrition. It is crucial to note that 3D printing imposes stringent performance of food gels, where rheological properties play a decisive role in the success of the 3D printing process [29]. Therefore, it is essential to gain a deeper understanding of the component interactions, the rheological performance of food gels, and their impact on the assembly of MAs.

This review critically discusses recent advances in food gelling systems for MAs and RMPs, with a focus on hydrogels, emulsion gels, oleogels, and hybrid gels; protein–polysaccharide hybrid gels; enzymatically crosslinked gels; and their integration with emerging structuring technologies like high-moisture extrusion, shear cell techniques, and 3D printing. Finally, this review summarizes the existing information to identify the trends, challenges, and future research requirements pertinent to academia and industry.

## 2. Gelation Ingredients

The growing acceptance of MAs and RMPs has led to increased incorporation of plant biomaterials, such as proteins, polysaccharides, and fats, as potential contenders for food gels. Understanding the gel-forming capacities of biomaterials is essential for the formulation of MAs. Food gels are three-dimensional crosslinked networks capable of immobilizing a large amount of water or oil within a continuous phase. In meat and meat analog systems, gelation can be driven by protein denaturation and aggregation, polysaccharide chain association, or lipid crystallization, and is stabilized by covalent bonds (e.g., disulfide, enzymatic cross-links) and non-covalent associations (hydrogen bonds, hydrophobic interactions, electrostatic forces) [30]. The main ingredients for gel formation are proteins, polysaccharides, lipids, their combinations, and enzymes, which are described in the following sections. The final gel properties are strongly influenced by biopolymer type, concentration, pH, ionic strength, temperature profile, and shear history [31].

### 2.1. Proteins

Given the varied use of protein gels in the MA and RMP sector, it is essential to determine the gelation mechanism and characteristics of both plant and animal proteins. Gelation of proteins imparts a fibrous nature to MAs/RMPs by forming a three-dimensional framework through protein–protein and protein–solvent reactions, yielding a slightly solid, viscous, and elastic structure. Gels can vary in firmness, transparency, and ductility based on the composition and source. Finally, the gel characteristics are significantly mediated by the concentration, pH, ionic strength, temperature, and shear parameters [30]. In animal proteins, gel formation begins when actin and myosin are heated and the Z bonds break down, allowing intramolecular water and fat to fill the gaps [32]. In MAs and RMPs, functional plant biomaterials like soy protein isolate (SPI), pea protein isolate (PPI), wheat gluten, oilseed extracts, etc., should be compatible with polysaccharide and fat biomaterials to be able to form a fibrous structure [33]. Moreover, plant-originated proteins sometimes lack structural quality and chemical characteristics compared to animal proteins, which may impede gelling and result in an unfavorable texture [34]. To overcome these concerns, researchers have modified the gelling technique and mechanism to develop MAs and RMPs with a physical appearance similar to that of conventional meat [35].

### 2.2. Polysaccharides

The gelation process of polysaccharides involves physical crosslinking via hydrogen bondsionic and hydrophobic interactions. With increased temperatures, polymers gather with hydrophobic domains and reduce contact with water molecules. Finally, gel formation occurs by molecular chain associations [36,37]. Several factors initiate the gel formation mechanism in polysaccharides, such as the addition of salt, acid, or alcohol. Each process illustrates unique gelling behavior and distinct traits, and increases its applicability in different products [38,39,40]. Polysaccharide gels are mostly formed using alginate, carrageenan, gellan gum, and konjac glucomannan due to their excellent water-binding and gelation characteristics [41].

### 2.3. Lipids

In MAs and RMPs, lipid-based gels serve as substitutes for animal fat, providing enhanced fatty acid compositions and melting qualities [42]. Emulsion gels and oleo gels serve as efficient carriers for encapsulating colors, flavors, antioxidants, nutrients, vitamins, and functional ingredients [43]. In this type of gel, lipid biomaterials are usually dissolved in the oil phase, while hydrophilic biomaterials are dissolved in the water phase. The rheological characteristics can also be further regulated in gels by adjusting their composition and structure [43,44].

## 3. Types of Gelling Applications in Meat Analogs and Restructured Meat

**Figure 1:**
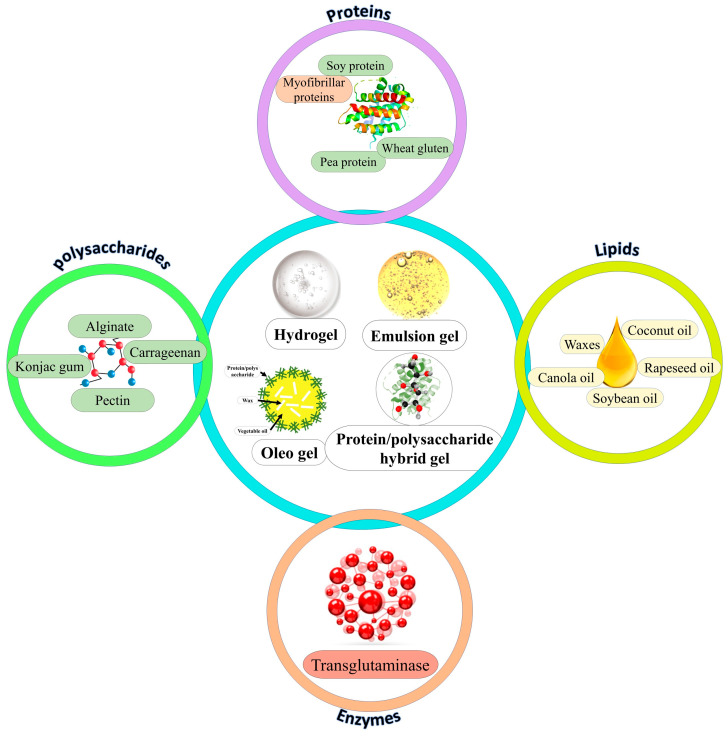
Types of gels and biomaterials used for developing gels for application in meat analogs and restructured meat products.

Given the types of MAs and RMPs and their manufacturing technologies, researchers have developed compatible gel types tailored to the molecular structure and gelation nature of the biomaterials [13,45,46]. Researchers have tested a wide range of biomaterials to develop various gels for efficient application in MAs and RMPs, as summarized in Figure 1. Moreover, the functional properties, uses, and limitations of these gel types have been detailed in Table 1.

### 3.1. Hydrogels

Hydrogels are defined as multilayered structures composed of crosslinked biomaterials suspended in a water-based solution, and this can be incorporated into the MA/RMP formula to enhance nutrition and texture profiles [47]. Hydrogels are known for their ability to mimic the texture and mechanical properties of animal fat, thereby improving the sensory and textural profile of MA products [48]. Plant proteins are recognized as key constituents for forming hydrogels owing to their superior hydration and gelation characteristics [49]. Another biomaterial commonly used in the production of hydrogel is polysaccharides due to their functional, thickening, emulsifying, gelling, and binding activities [50].

Various protein types can be used to produce hydrogels depending on their solubility in water or an aqueous medium, including animal, plant, and engineered proteins [51]. Plant-derived protein biomaterials possess exceptional solubility in water, are able to form superior gelling, and are thus more preferred in hydrogel production [49]. Nevertheless, a prevalent concern with pure plant protein hydrogels is their excessively soft and fragile nature, which makes them susceptible to rupture under minimal pressure and unable to mimic the fibrous characteristics of real meat [52]. To address this concern, new studies are being conducted to improve the functionality of hydrogels by modifying their architectural network. Hu et al. [53] fabricated imitated MA salami and pepperoni with intramuscular fat with hybrid alginate hydrogels combined with potato protein and gellan gum. The structural integrity of the MAs varied with higher protein content and protein type. Incorporation of a hybrid hydrogel under compression conditions has minimal effect on its textural profiles, but stiffness was reduced. A separate experiment assessed the effect of cellulose incorporation on the microstructure and textural profile of potato protein (20%) hydrogels. It indicates that this hydrogel can be applied to MAs and RMPs to improve the textural profile and physical appearance [54]. Zhao et al. [22] constructed an interconnected polymer network hydrogel comprising crosslinked SPI, transglutaminase (TG), and microbial polysaccharide. In this experiment, the incorporation of microbial polysaccharides modified the secondary structure of SPI, offering an efficient method to enhance the quality of MAs by improving structural integrity during refrigeration. The latest research on MAs investigated emulsion gels made from fat-infused RuBisCo protein hydrogels [44]. The outcomes demonstrated that increasing the protein content in the gels precisely mimicked the water-retention quality of cooked chicken meat. Biochemical changes in meat muscle usually affect water retention and variation in cooking-related losses and shrinkage. A similar mechanism was applied to the MA in this experiment. An investigation showed pea protein in hydrogels is effective at masking flavors and odors generated by the incorporation of linseed oil [55]. Pea protein hydrogels at 5% and 10% oil content reduced sensory limitations, with 10% demonstrating particularly advantageous qualities, notably in terms of textural profiles due to the fat content.

Polysaccharides are frequently incorporated as a basic biomaterial for the production of hydrogels [56]. Utilizing plant-based polysaccharides is a highly efficient method to improve the structural and functional attributes of hydrogels, owing to the superior gelling, binding, and emulsification activities of polysaccharides [50]. Zhou et al. [52] examined the influence of table salt, pectin, and TG on the morphology and gelling ability of protein extracted from potato. The findings indicated that incorporating salt can enhance the structural integrity of protein hydrogels by minimizing electrostatic interactions; however, TG may increase gel strength due to its better crosslinking ability. The incorporation of pectin reduced the resilience of the gel but enhanced its strength and increased its elasticity, porosity, and the waviness of its structure. Researchers have modified pea protein with β-glucan, altering the hydrogel’s microstructure and integrity via temperature regulation, thereby creating a diverse structure that is not achievable with conventional extrusion methods [57]. The incorporation of alginates enhances both the structural and mechanical strength of pea protein hydrogel while also facilitating water retention [58]. Chen et al. [59] produced hybrid hydrogels using glucomannan and carrageenan as a fat replacement for frankfurters. However, substitutions of 60% or more caused reductions in the textural integrity and taste of the frankfurters. Consequently, meticulous evaluation is essential when employing hydrogels as a fat replacer to ensure that MA quality and taste are not compromised. Flavor is a significant limitation to consumer acceptance of MAs and RMPs, which can be addressed by encapsulating flavor with alginate hydrogel. Alginate hydrogel compatible with MAs was produced by combining various polysaccharides by Keum et al. [60], where β-cyclodextrin significantly enhanced the encapsulation of taste by 91.78%. Four different hydrogel integrations in MA patties enhanced the quality parameters and chromatic and textural profiles, and reduced cooking-related losses over four days of refrigerated preservation.

### 3.2. Oleogels

Oleogels are semi-solid food gels prepared by the combination of plant oils and food-grade oleogelators. Oleogelators are formed by structuring liquid oils into a gel by using a solid to encapsulate the oils [61]. Oleogelators can self-assemble into oleogel networks within the oil matrix. Oleogels are widely used in various food products to improve their sensory profile. Oleogels comprise monoglycerides, fatty acids, fatty alcohols, waxes, polysterols, esters, and ethylcellulose [23]. MAs and RMPs consist of plant-derived proteins, lipids, and carbohydrates, along with additives to imitate conventional meat characteristics [62]. Fat is an essential component to imitate the textural profile, taste, and juiciness of meat. Thus, MAs and RMPs contain up to 15% fat [63]. The inclusion of unsaturated fatty acids in MAs and RMPs may contribute to off-flavors due to oxidation metabolites during either manufacturing or cooking [62]. Semi-solid oleogels are produced from vegetable oils or oleogelators [44]. They provide a multifaceted approach to structuring oils and improving the sensorial characteristics of MAs and RMPs. Moreover, integrating oleogels as a fat replacer in MAs provides a healthier meat alternative for customers. Consequently, substituting animal fat with oleogels is an effective approach to replace saturated fats while maintaining the solid structure and nutritional properties of MAs [64]. The integration of oleogels exhibited excellent oil-binding capability and superior textural properties, particularly for MAs and RMPs. Li et al. [23] examined the physical and chemical characteristics of canola oil oleogels separately blended with rice bran oil, beeswax, candelilla wax, and carnauba wax to prepare MA burger patties. The results indicated that incorporating this oleogel enhanced the texture, taste, and flavor characteristics of the patties. Oleogels composed of candelilla wax have been proposed as the ideal blend for MA patties.

Flores et al. [65] examined the impact of sunflower oil oleogels blended with hydroxypropylmethylcellulose and xanthan gum on their incorporation in MA patties. These oleogels enhanced the fatty acid profile of the MA patties, decreased cooking-related losses, and enhanced the chromatic and textural profiles of the MA patties. Huang et al. [66] investigated the efficiency of different vegetable oil-based oleogels in the 3D printing of MA products. They used soybean, canola, coconut, and palm oils to produce oleogels. The findings indicated that palm and coconut oil oleogels showed superior rheological characteristics and performed efficiently in 3D printing.

### 3.3. Emulsion Gels

Emulsion gels are created by incorporating an emulsion into a gel structure [67]. There are two phases in an emulsion gel: dispersed phase (emulsion) and continuous phase (gel). Usually, emulsion gels demonstrate unique water retention ability and a resilient structure compared to other gels [42]. Emulsion gels are pliable solid substances that integrate the combined benefits of emulsions and gels. They may exhibit semi-solid properties due to the presence of a polymer matrix in the liquid phase and droplet aggregation [67]. Due to their multifaceted benefits, emulsion gels are preferred for MA and RMP production today. Significant focus has been placed on emulsion gels in MA and RMP formulations to fabricate low-fat, sugar-free, and salt-free products to ensure consumer health [67]. Emulsion gel application encompasses the formulation of low-fat products by reducing sugar and salt levels in MA products. Another application of emulsion gels is the encapsulation of probiotics, essential oils, and functional biomaterials for timed release in the intestine, thereby prolonging their activity [67]. Emulsion gels can enhance the flavor profile and textural characteristics of MAs and RMPs [31]. The research on emulsion gels for MAs and RMPs encompasses an extensive investigation of their processing parameters and characteristics, employing various plant proteins to comply with the characteristics of conventional meat [44]. Emulsion gels exhibit the ability to modify different rheological parameters to ensure the required sensorial profile and minimize the off-flavors of plant biomaterials to satisfy MA and RMP consumers [68].

Botella-Martinez et al. [69] developed an RMP burger patty using emulsion gels and found no deviation in ash level, but variations in iron level were observed between the control and emulsion-supplemented RMP, depending on whether chia seed or hemp seed oil was used. The noted variation in iron level may be ascribed to the elevated moisture levels in the RMP patties.

MA frankfurters made with oats and flaxseed oil emulsion gels exhibited no significant variations in ash content when compared to conventional meat frankfurters [70]. Moreover, the crude protein level of the oat group was comparable to that of the control sample, but the flaxseed group had lower levels. However, flaxseed-based sausages exhibited a significantly lower protein content. Nevertheless, the control group had a markedly elevated fat content, likely due to the prevalence of unsaturated fatty acids in meat. In another experiment, emulsion gels prepared by incorporating ribulose 1,5-bisphosphate carboxylase protein mimicked the physical, chemical, and textural properties of chicken breast [44]. The application of emulsion gels in MA sausages led to notable variations in chromatic profiles (lightness and redness) compared to meat sausages [70]. Chromatic variations were probably associated with the incorporation of spices (red paprika). The elevated lightness in MA samples may be due to greater light dispersion from the oil droplets with increased diameters [70]. Botella-Martinez et al. [69] investigated the textural profiles of MA patties incorporating emulsion gels. This study revealed no notable variations in textural profiles (springiness and chewiness) across all patty samples. This study also investigated the integration of beetroot juice in MA patties and found a higher shear force, while emulsion gels did not interfere with the textural parameters. These results demonstrate that the structural parameters of MA patties are affected by the type of emulsion gel along with other additives. Tan et al. [44] demonstrated that the rheological parameters of emulsion gels may be meticulously adjusted by modifying the protein or oil concentrations to alter the textural profiles of MAs and conventional meat products. More extensive studies are required to understand the relationships among MAs and RMPs and emulsion gels made from various plant-based biomaterials.

### 3.4. Hybrid Gels for Meat Analogs

A hybrid gel can be defined as a gel that is produced with different combinations of gel-forming biomaterials like gelatin, collagen, chitosan, soy protein, pea protein, cellulose, starch, or alginate incorporated with functional biomaterials to improve their mechanical strength [71]. It is challenging to fabricate plant-based food gels and to apply them to develop MAs and RMPs that mimic the characteristics of conventional meat [72], due to higher moisture and lower hardness [73]. Hybrid food gels can rectify these limitations by functioning as bio-glue, enhancing the chromatic profile, physical properties, and taste traits of MAs and RMPs. Different combinations of hybrid gels have been discussed in Table 2. Moll et al. [74] conducted a study to develop a multiple-function hybrid gel incorporating pea and red beet protein with cellulose to improve the binding capacity, chromatic profile, and structural characteristics of MA patties. This hybrid gel optimized the processing parameters of extrusion techniques for MA and RMP production, resulting in a better textural profile and juiciness. Sun et al. [75] produced another hybrid gel containing SPI, coconut oil, potato starch, and glucomannan to investigate the binding characteristics of the gel and MA. This hybrid gel efficiently performed as a bio-glue, enhancing the rheological parameters, structural integrity, and cooking qualities of the MA. This was achieved through effective crosslinking between starch and glucomannan. Protein–polysaccharide hybrid gels were developed by gelation of potato protein and sodium alginate in calcium chloride solution to study their rheology and structural integrity [76]. This hybrid gel was proven appropriate for the production of MA and RMP on large scale.

**Table 1:** Different gel applications in meat analogs and restructured meat products.

Gel Type	Biomaterials	Primary Role	Principal Benefits	Limitations	References
Protein hydrogel	Gelatin, soy protein, pea protein, wheat gluten, myofibrillar proteins, microbial protein	Binder, structural development, and moisture retention	Better mechanical integrity, availability of biomaterials	Brittleness, weak texture compared to animal proteins	[77,78,79,80]
Polysaccharide hydrogel	Alginate, pectin, carrageenan, gellan, konjac gum	Thickener, moisture retention, structural retention, thermal stability	Adjustable gelation, clean-label, low production cost	Limited elasticity; requires cogelators	[81,82]
Emulsion gel	Vegetable oils, plant-based waxes	Fat replacer, lubricant, encapsulation of flavor materials	Enhanced fatty acid profile, improved and controlled melting	Oxidative instability, off-flavor	[82,83,84]
Oleogel	Ethylcellulose, proteins, sesame oil, olive oil	Fat replacer, lubricant, encapsulation of flavor materials	Better texture, controlled melting	Oxidative instability, off-flavor	[85,86]
Hybrid gel	Combination of all gel types	Imitation of fat tissue, controlled release of hydrophilic or lipophilic nutrients	Customizable, intricate sensory characteristics	Stability, scalability	[87,88]
Enzymatically and chemically crosslinked gels	Transglutaminase, Laccase	Binder, structural development, bio-ink preparation for 3D printing	Improvement in textural and chromatic profiles	Stability, cost	[89,90]

**Table 2:** Hybrid gel strategies for meat analogs and restructured meat products.

Protein	Polysaccharide	Cross-Linking Technique	Performance	References
Soy protein	Methylcellulose, carrageenan	Heat-induced gelation, thermoreversible setting	The hybrid gel system improved the textural profile, particularly in terms of hardness and chewiness. Furthermore, the cooking-related losses were decreased.	[91,92]
Pea protein	Pectin, xanthan, guar gum	High-pressure treatment, heat-induced gelation	Increased storage modulus, ability to form smooth emulsion, and better structural integrity	[92,93]
Wheat gluten	Alginate, glucomannan	Calcium crosslinking, baking	Better sliceability and elasticity in whole-cut analogs	[13]
Mixed legumes	Gellan gum, locust bean gum	Enzymatic crosslinking (MTGase)	Strong composite network, high water-holding ability, improved freeze–thaw stability	[79]

### 3.5. Crosslinked Gels

Crosslinking in food gel making is the process of forming covalent bonds between different biomaterials during their polymerization. This is achieved by introducing a specific crosslinking agent, such as a chemical or enzyme, or by physical force, such as heat or ultrasound. Crosslinking for gel preparations is critical to serve as a binder in the MA/RMP production process [94].

In MA or RMP development, vegetable oils are commonly used as fats, such as coconut, sunflower, canola, and rapeseed oil, but they have limitations compared to animal fat in terms of mechanical characteristics [95]. Food gels have shown efficacy to imitate the characteristics of animal fat using modified vegetable oil emulsion gels with the TG enzyme [93]. This straightforward approach facilitates the development of MA and RMP from plant proteins by combining them with enzymatic crosslinking to improve their binding and texture profiles. Zhou et al. [52] conducted an experiment to examine the effects of sodium chloride, pectin, and TG on the binding and structural profiles of potato protein gels to develop MA products. The incorporation of sodium chloride enhanced the gel strength by diminishing interactions during gel formation. Moreover, TG enhanced the gel porosity and diminished the structural strength, but these characteristics ultimately resulted in an MA with a fibrous structure. Herz et al. [96] produced MA salami using glucono-δ-lactone with and without TG to form SPI gel, thereby improving the binding efficiency of the products. Furthermore, this gel facilitated the imitation of aged sausage by incorporating additional fat molecules. A further study sought to use gels made from camellia seeds as a carrier for fat integration within textured vegetable protein and wheat gluten. This hybrid gel was further crosslinked with TG and sodium alginate to fabricate porous gels, yielding MA products with improved textural and chromatic profiles, especially reduced redness [90]. Heat-induced gels are mainly used as a gelation technique for plant proteins. Heat causes unfolding of the plant proteins into a simpler form to retain water, oil, and emulsion systems, and enhances the structure of the MA/RMPs [2]. The water retention, viscoelastic, and mechanical features of food gels can be significantly improved by using heat-induced gels. The literature confirms that the integration of heat-induced gels into fava starch and cassava starch hybrid gel enhanced their texture profile, especially the chewability [97]. Another technique is ultrasound, an innovative, green crosslinking method to enable the scalable production of food gels. Xu et al. [98] reported that ultrasound combined with heating improved the gelatinization, gel properties, texture, and microstructure when the gel was used as an ink for 3D printing.

Crosslinking for gel preparations is evolving as an efficient technique in the MA/RMP production process. Among different methods, TG remains the predominant bio-glue for efficient production of plant-based biomaterial gels and hybrid gels. Future investigations should focus on the application of crosslinked gels for the scalability of HMET and 3D printing technology, aiming to produce MA/RMP in a scalable manner that mimics conventional meat properties.

## 4. Structuring Technologies Integrating Food Gels

Modern MA processing techniques can activate food gels to enhance binding, fat and flavor incorporation, and imitate a fibrous structure in MAs and RMPs. Hydrogels, emulsion gels, hybrid gels, and crosslinked gels can be developed alongside scaffolding materials by using enzymatic and chemical crosslinking techniques. These modifications facilitate gels, thereby improving the physicochemical, textural, and flavor characteristics of MAs and RMPs [91]. The fundamental concept of integrating structuring technologies with food gels is to develop MAs and RMPs with a diversified gel network, smooth bio-ink with improved rheology, to facilitate pattern printing, and, furthermore, to mimic the structural and taste characteristics of conventional meat products [42].

### 4.1. High-Moisture Extrusion Technology

High-moisture extrusion technology (HMET) is a technique for extruding plant materials with more than 40% moisture to a fibrous structure. This method is unique for its precise cooling of post-melt dough. The final MA product in HMET is an enlarged long fiber due to the exertion of steam generation [99]. Researchers recommend HMET as a sustainable and environmentally friendly method for producing MA from plant-based protein biomaterials [100].

HMET is an extremely advanced and recognized method for the scalable production of MAs and RMPs with a fibrous texture that imitates conventional meat. In the HMET method, plant proteins are heated to melt, then passed through screw extruders to develop fibrous structures that imitate the structural characteristics of conventional meat muscle [101]. Food gel formation is an essential element of structured MA production in HMET. Plattner et al. [102] reported, in their study using pea protein and soy protein in the HMET technique, that there is a positive relationship between the protein type and gel strength in forming fibrous MA products. Furthermore, food gels can be integrated into HMET to improve the structure, flavor, and color profile of the MAs and RMPs. Different hydrocolloids, such as glucomannan, methylcellulose, and carrageenan, are combined with protein biomaterials to calibrate the viscosity, water retention, gel strength, and other rheological parameters during the cooling step. Furthermore, these hydrocolloids assist in the gelation process, produce myofibril-like long, continuous imitated meat fibers, and alleviate dryness [13]. In another study, Liu et al. [103] described how different gel types affect the extrusion process during HMET during MA product fabrication. They showed that incorporating emulsion gels increased the heat stability and maintained a stable tertiary structure of soy protein compared to polysaccharide gel and hybrid protein–polysaccharide gel during the extrusion process. Moreover, texturization at high temperature was more stable with emulsion gel than with polysaccharide gels. Emulsion gels with different oil blends can be incorporated into the protein and polysaccharide matrix to fabricate MAs and RMPs with improved hardness, chewiness, and juiciness. Moreover, they can imitate the intramuscular fat of conventional meat structures [104]. Enzymatically crosslinked gels can be incorporated during or after the extrusion process to enhance the structural integrity and shear quality of the MAs and RMPs [42]. The effective integration of different types of food gels into MA and RMP manufacturing techniques can ensure the necessary physicochemical, color, textural, and sensory characteristics while using HMET [105]. Future research on HMET should focus on the gelling chemistry, kinetics, rheological parameters, and practical applications in MA, RMP, and cultured meat (CM) development. Furthermore, the mechanical configuration of HMET machines is important to study, including the screw specifications, temperature, and moisture, and the integration of machine learning algorithms to produce imitated meat products [33].

### 4.2. Shear Cell Technology

The shear cell technique combines heat and mechanical processing to fabricate fibrous MA products. It is known as a modification of the HMET technique [106]. This process consists of two flat or conical plates, of which one is fixed and the other rotates to apply shear. It is possible to imitate a conventional meat-like fibrous structure with the application of the high-temperature shear cell technique [106,107].

**Figure 2:**
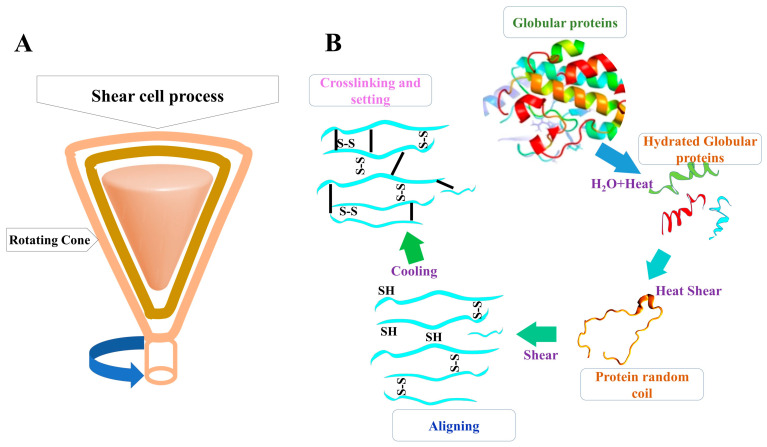
Shear cell technology for food gel production; (**A**) shear cell process outline; (**B**) basic chemical process in shear cell technique.

Shear cell is a novel economical technique that enables reduced-energy batch processing of fibrous and layered MAs and RMPs with the application of geometrics, such as cone-like cylindrical devices. Imitated conventional meat-like fibers can be produced with the high-temperature shear cell technique, which involves two rotating flat or conical plates to apply shear [106]. Compared to HMET, this method requires a higher temperature and a longer processing time, facilitating the production of aligned imitated fibers through controlled gelation [91,108]. The shear cell technique is more convenient, with a well-defined shear field and temperature distribution [109]. The chemistry behind the shear cell technology is illustrated in Figure 2.

It has been observed that integrating food gel enhances the structural characteristics of MAs and RMPs during the shear cell technique. The integration of protein–polysaccharide hybrid gel materials during cooling facilitates the development of the gel network and results in a solidified, aligned fibrous structure that resembles a conventional meat structure. In the shear cell technique, the application of gels made from pectin, gellan, and xanthan can efficiently modify the viscosity and gelation characteristics to produce aligned fibers with flexibility [13,108,110]. Krintiras et al. [111] developed a MA in a Couette cell by applying simple shear flow and heat to develop an MA with an aligned fibrous structure resembling conventional meat. This process in the experiment was enhanced by using SPI- and PPI-based gel complexes. Sägesser et al. [107] observed similar characteristics during their development of aligned MA fibers using a high-pressure shear cell technique. They concluded that SPI and PPI hydrogels enhance the viscosity and texturization parameters in the shear cell process.

### 4.3. 3D Printing Technology

**Figure 3:**
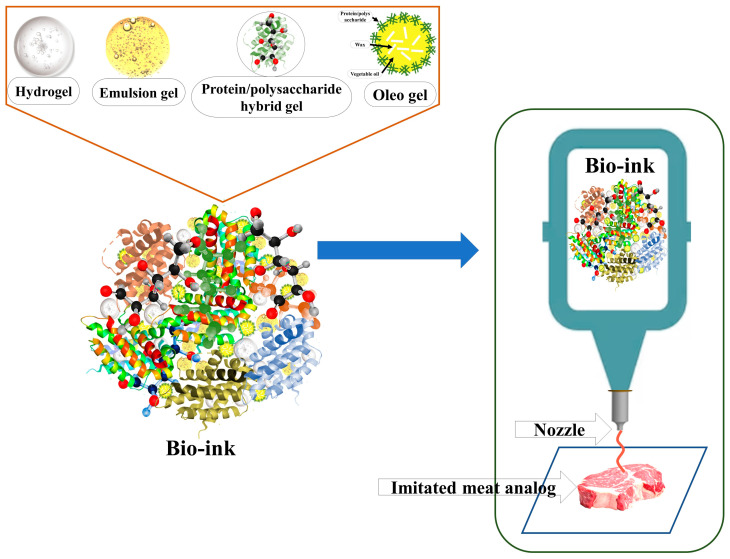
Application of food gels in bio-ink in the 3D printing of meat analogs.

A conventional meat structure can be imitated using 3D printing technology. This technique comprises computer programs, artificial intelligence, mechanical and mathematical control, and material engineering [112]. In the 3D printing technique, plant-based or hybrid inks are deposited in layers with controlled rheological and gelation parameters for the production of specific meat cuts of MA or CM products [113]. Food gels are essential components of 3D printing formulations in multiple dimensions. In MA and RMP production, plant-based or hybrid bio-inks are generally used (Figure 3), which are mostly protein–polysaccharide hydrogels or emulsion gels, as they facilitate extrusion and maintain structural integrity [114].

Hydrogels and emulsion gels composed of SPI, PPI, mycoprotein hydrogels, and emulsion gels blended with methylcellulose, alginate, or xanthan are used as 3D printing inks as they demonstrate shear-thinning properties and regain their macromolecular structure immediately after deposition [91]. 3D CMs or MAs are a relatively recent technique; nonetheless, various studies have employed diverse methodologies to produce real-cut MA or CM products. Park et al. [115] and Lee et al. [116] developed imitated meat structures using 3D printing with surimi gels integrated with plant protein, beef paste, and fat as a hybrid ink. Emulsion gels and hydrogels have been shown to improve the fluid dynamics of inks during layered deposition, thereby ensuring a fibrous, meat muscle-like structure [35,89]. Ko et al. [117] employed a hybrid gel comprising SPI, potato starch, sodium alginate, glucomannan, and carrageenan for incorporation into ink for the 3D printing of MA. In this experiment, coaxial extrusion facilitated the deposition of fibrous structures. Future studies should focus on understanding the dynamics of the combined use of emulsion gels, fiber-rich gels, and hybrid gels in 3D printing conditions and how they ensure the layered deposition of an imitated meat structure. Furthermore, extensive research is needed to assess the sensory and textural profile of MAs and RMPs, while using gel-based inks to ensure scalability.

## 5. Significance of Gelling Processes on the Sensory and Nutritional Components

Substantial research has been conducted on investigating the application of food gels in MA and RMP development. Their application encompasses the development of a utility that extends to the creation of low-fat, salt-free, and sugar-free diets and the integration of probiotics to enhance consumers’ intestinal health [67]. Moreover, food gels may be crucial for achieving imitated sensory quality and textures while concurrently improving physical stabilization. Food gels, including plant essential oils, represent a feasible substitute for animal fats in MA and RMP production [33]. Encapsulation of color, flavor, nutraceuticals, and functional ingredients in food gels may enhance their capacity to improve the intended quality and nutritional profile of MAs and RMPs. To ensure sustainability and economic viability, food gel research encompassing thorough investigation of gelling research mechanisms spans the use of diverse plant proteins to cater to the needs of the MA and RMP production process [44]. PPI has emerged as a significant option for the formulation of food gels to develop MAs, CM, and RMPs to imitate conventional meat [118,119]. Moreover, heat-induced hydrogel prepared in combination with PPI and SPI demonstrated superior structural strength and was deemed suitable for MA production [120]. Innovative approaches such as ultrasound, microfluidization, homogenization, and pH adjustments are being investigated to augment the functional properties of PPI hydrogels, improve gelling properties, and improve the nutritional profile of MA products [121,122].

Hydrogels, emulsion gels, and hybrid gels play a crucial role in the sensory profile of MA and RMP. The sense of juiciness is closely associated with the release of fluids during chewing. The juiciness perception is strongly linked to the release of serum during chewing, which is dependent on the binding of water and oil within the gel composition. Compact gelling can retain moisture in the MA, but less exudate is formed within the package, which lowers the juiciness. Whereas, a hybrid of more loose gels can cause fluid release and ensure juicier MA products [79]. Research comparing MA burger patties with different gel compositions indicates that optimum gelation can be enhanced. Studies comparing plant-based burgers with different hydrocolloid systems show that optimized gelation can increase juiciness, softness, and ultimately consumer satisfaction. Emulsion gels and oleogel in MA patties enhance mouthfeel by increasing water and oil retention, imitating animal fat [42]. Food gels also influence flavor perception by binding taste traits through encapsulation of functional compounds, regulating their release during cooking and consumption. Hybrid protein–polysaccharide gels can further enhance the distribution of water and oil, regulating fluid release and improving the taste and textural profile of MAs and RMPs [66].

## 6. Limitation, Research Gaps, and Future Directions

Although substantial progress has been made in food gels for incorporation in MAs and RMPs, certain challenges limit practical application.

The majority of research emphasizes physical, chemical, textural, sensory, and storage studies conducted on a short-term basis at the laboratory scale. Furthermore, gels derived from plant biomaterials exhibit an incomplete amino acid profile compared to animal-derived biomaterials, which may pose a risk of deficiencies in specific amino acids such as lysine and methionine, among consumers of MAs or RMPs. Moreover, essential elements like vitamin B12 and omega-3 and 6 fatty acids, often abundant in animal-originated gels, are sometimes insufficient in plant-based gels, and thus require supplementation to fulfill the compatibility of MAs with conventional meat. In addition, clinical trials and consumer studies are limited to validate health claims and acceptability from a practical perspective. The rheology of food gels and gelling materials substantially influences the qualitative properties of MA, especially the textural profile. They are significantly influenced by different processing parameters, including density, shear rate, and temperature. It is essential to accurately specify the rheological features of the used biomaterials and the final textural profiles during various MA production techniques. Machine learning is a part of artificial intelligence and a rapidly evolving technique for enhancing the predictability of patterns using algorithms and software which should be incorporated in MA research and development [123].

From a technical perspective, food gels face challenges in scalability and economic viability due to energy-intensive extrusion and HMET. From an economic perspective, 3D printing can optimize raw material consumption, minimize waste, and simultaneously imitate real meat structure.

Therefore, extensive research on 3D printing should be conducted, focusing on the rheological standardization of the bio-inks. Emphasis should be placed on the formulation of emulsion gels, hydrogels, and hybrid gels using plant and agricultural food waste biomaterials to reduce cost and environmental waste.

## 7. Conclusions

The use of various gel types in MAs and RMPs has demonstrated mostly positive results, consistent with quality parameters such as enhanced water retention, moisture content, reduced cooking-related losses, and the achievement of textural and sensory attributes. Despite comprehensive research on food gels, there are few publications on their application to MA, RMP, and CM products. While recognition in this context is gradually increasing, there is a need for further research to assess the feasibility of different agricultural food waste-based biomaterials for gelling and incorporation into MAs and RMPs. This will ensure environmental sustainability by reducing greenhouse gas emissions. Additionally, there is a need for an in-depth investigation into the nutritional, sensory, and physicochemical characteristics of MAs supplemented with various food gels, particularly the sensory attributes influencing product success and consumer preference. From a regulatory perspective, the preparation of food gels for MAs and RMPs is primarily based on approved raw materials, as in the food industry [124]. This is an added benefit of obtaining easy permission from the regulatory authorities in different countries. However, it is essential to define, characterize, and obtain approval for labeling, classification systems, and related laws for MAs and RMPs to ease global market development. In summary, food gels hold considerable promise due to their adaptability, which enables a wide range of applications, including the substitution of fats and proteins for producing MAs and RMPs with improved nutritional profiles. As research on food gels advances, the industry expects MAs and RMPs that properly imitate the textural and sensory profiles of conventional meat, satisfying consumers seeking sustainable options. Nonetheless, artificial intelligence can substantially improve the processing and production of MAs and RMPs that precisely imitate conventional meat and meat products. Upcoming investigations should prioritize the diverse application of this technique to improve the scalability of MA and RMP production with improved textural qualities.

## Data Availability

No new data were created or analyzed in this study. Data sharing is not applicable to this article.
